# Numerical Simulation of Corneal Fibril Reorientation in Response to External Loading

**DOI:** 10.3390/ijerph16183278

**Published:** 2019-09-06

**Authors:** Dong Zhou, Ahmed Abass, Ashkan Eliasy, Alexander Movchan, Natalia Movchan, Ahmed Elsheikh

**Affiliations:** 1 School of Engineering, University of Liverpool, Liverpool L69 3BX, UK (A.A.) (A.Eli.) (A.Els.); 2 Department of Mathematical Sciences, University of Liverpool, Liverpool L69 7ZL, UK (A.M.) (N.M.); 3 NIHR Biomedical Research Centre for Ophthalmology, Moorfields Eye Hospital NHS Foundation Trust and UCL Institute of Ophthalmology, London EC1V 9EL, UK; 4 School of Biological Science and Biomedical Engineering, Beihang University, Beijing 100191, China

**Keywords:** tissue microstructure, numerical modelling, ocular biomechanics

## Abstract

Purpose: To simulate numerically the collagen fibril reorientation observed experimentally in the cornea. Methods: Fibril distribution in corneal strip specimens was monitored using X-ray scattering while under gradually increasing axial loading. The data were analysed at each strain level in order to quantify the changes in the angular distribution of fibrils with strain growth. The resulting relationship between stain and fibril reorientation was adopted in a constitutive model to control the mechanical anisotropy of the tissue material. The outcome of the model was validated against the experimental measurements before using the model in simplified representations of two surgical procedures. Results: The numerical model was able to reproduce the experimental measurements of specimen deformation and fibril reorientation under uniaxial loading with errors below 8.0%. With tissue removal simulated in a full eye numerical model, fibril reorientation could be predicted around the affected area, and this change both increased with larger tissue removal and reduced gradually away from that area. Conclusion: The presented method can successfully simulate fibril reorientation with changes in the strain regime affecting cornea tissue. Analyses based on this method showed that fibrils tend to align parallel to the tissue cut following keratoplasty operations. With the ability to simulate fibril reorientation, numerical modelling can have a greater potential in modelling the behaviour following surgery and injury to the cornea.

## 1. Introduction 

Soft tissue has an important role to bind and structurally support the components of the human body using an extracellular matrix (ECM) composed of proteins, glycosaminoglycan and water [1]. The ECM, and in particular its collagen fibrils, provides the tissue with mechanical stiffness to withstand external and internal loads. For this reason, considering collagen fibrils and their effect in determining the level, regional variation and anisotropy of stiffness, is becoming essential in numerical simulations of ocular mechanical behaviour [2,3,4,5,6,7,8,9,10].

A more recent challenge has been the growing evidence that the orientation of fibrils is not static but changes with variations in the strain, to which the tissue is subjected, which typically arise due to surgery, injury or disease [11,12,13,14]. The process of fibril reorientation should, therefore, be considered if the effect of these events was to be simulated accurately [15,16,17,18]. The literature includes reports of changes in fibril orientation around the optic nerve head in glaucomatous eyes [19], in the cornea with the progression of keratoconus [13,20] and in the area at which the keratoplasty took place [11].

These changes are believed to be due to the biosynthesis and degradation in tissue with cells modulating their behaviour according to alterations in their biomechanical and biochemical environment [14,21], and reorientation of fibrils also takes place in response to the same alterations [22,23].

Several biomechanical models have been developed to account for changes in the microstructure characteristics of soft tissues and represent the resulting changes in tissue’s mechanical response to load. Most models considered fibril remodelling (or volume-fraction-based reorientation) as a response to the mechanical stimuli caused by changes in either stresses or strains [18,24,25]. However, to the best of our knowledge, there has been no published attempt to consider simultaneously the changes in fibril quantity in multiple orientations with changes in strain or stress regimes. Driessen, et al. [26] considered only two fibril orientations, which changed based on the assumption that fibril preferential orientations aligned with principal strain directions. One year later, the same group extended their method to consider the rotation from four axisymmetric fibril orientations towards preferential orientations, which were situated in between the principal strain directions [27]. Alternatively, Hariton, et al. [28] considered only two directions and altered the angle between them based on the assumed positive relationship between the angle and the ratio of principal stresses. Meanwhile, Grytz and Meschke [18] and Driessen, et al. [29] used two fibril directions and one dispersion factor to represent fibril distribution. Fibril distribution was remodelled by updating the two fibril directions between principal stress directions and considering the change in dispersion factor when subjected to the difference between the principal stress values.

This study presents a step forward in seeking to simulate the simultaneous changes that can take place in fibril quantity at any point in 16 equally-spaced orientations. It also builds on an earlier step where experimental measurements were taken of fibril density and orientation across strips of corneal tissue subjected to uniaxial loading [22]. That step not only presented clear evidence of fibril reorientation with strain changes, it also provided data that has been employed in this study to quantify the gradual fibril reorientation and simulate it in a finite element nonlinear numerical model of ocular tissue. The data further enabled validation of the model and hence its reliable use in the simulation of the effects of injury and surgeries including keratoplasty on corneal biomechanical behaviour.

## 2. Methods

### 2.1. Material Constitutive Model

The material model considered the nonlinear and anisotropic material behaviour using a strain energy function. Isochoric split into volume changing behaviour and volume distortional behaviour was performed on the energy function. The volume changing behaviour concerned the material deformation that led to density change. Decupled from the material volume changes, the volume distortional behaviour was subjected to deformation of collagen fibrils and the ground substance matrix of ocular tissue. Therefore, the material model adopted the strain energy function with 3 components for volume dilation ($W_{\mathrm{vol}}$), collagen fibrils (${\bar{W}}_{A})$ and ground substance matrix $({\bar{W}}_{m}$):

(1) $$W=W_{\mathrm{vol}}\left(J\right)+{\bar{W}}_{A}\left(I_{4},I_{6},I_{3}\right)+{\bar{W}}_{m}\left(I_{1},I_{3}\right)$$

where J is the determinant of deformation gradient F, the third invariant $I_{3}$ = det(C), the first invariant $I_{1}=\mathrm{tr}\left(C\right)$. $C=F^{T}F$ is the Right Cauchy deformation gradient. The other invariants $I_{4}=a_{0}Ca_{0}$ and $I_{6}=b_{0}Cb_{0}$ represent the stretch of fibril in a direction specified by $a_{0}$ or $b_{0}$.

The component $W_{\mathrm{vol}}\;\left(J\right)$ was relevant to the volumetric changes and controlled the extent to which the material can be compressed under deformation:

(2) $$W_{\mathrm{vol}}\left(J\right)=\frac{1}{D}{\left(J-1\right)}^{2}$$

where D is given a small value ($10^{-5}\;$) to ensure the material’s incompressibility.

Verified by Studer, Larrea, Riedwyl and Buchler [9], the Neo-Hookean law was chosen to model the isotropic behaviour of ground substance matrix (${\bar{W}}_{m}$):

(3) $${\bar{W}}_{m}\left(I_{1},I_{3}\right)=C_{10}\left(I_{3}^{-1/3}I_{1}-3\right)$$

where $C_{10}$ is a material parameter describing the stiffness of the ground substance matrix.

The anisotropic part of strain energy (${\bar{W}}_{A}$) was modelled by considering in-plane (${\bar{W}}_{\mathrm{Lam}}$) and out-of-plane fibrils (${\bar{W}}_{\mathrm{Int}}$). In-plane fibrils are all aligned in the tangent-plane to the ocular surface. The angle between out-of-plane fibrils and tangent-plane fibrils was taken as 15° as reported in earlier studies [4,9]. A weighted average sum method was used to add strain energy contributions from fibrils at different orientations by a discretised fibril density function K:

(4) $${\bar{W}}_{A}\left(I_{4},I_{6},I_{3}\right)=\frac{1}{N}\overset{N}{\underset{i=0}{\sum }}Κ\left(r,\varphi ,\alpha ,\theta_{i}\right)\left({\bar{W}}_{\mathrm{Lam}}\left(I_{4},I_{3}\right)+{\bar{W}}_{\mathrm{Int}}\left(I_{6},I_{3}\right)\right)$$

where N is the number of discretised fibril orientations and $Κ\left(r,\varphi ,\alpha ,\theta_{i}\right)$ is the fibril density calculated based on the global spherical coordinates of the material point (r,φ,α) and the local angle of orientation $\theta_{i}$. On the other hand, ${\bar{W}}_{\mathrm{Lam}}$ and ${\bar{W}}_{\mathrm{Int}}$ took the same form as that proposed by Markert, et al. [30]:

(5) $${\bar{W}}_{\mathrm{Lam}}\left(I_{4},I_{3}\right)=\frac{\mu_{1}}{\gamma_{1}}\left(\left(I_{3}^{-1/3}I_{4}\right){\;}^{\frac{\gamma_{1}}{2}}-1\right)-\mu_{1}\ln{\left(I_{3}^{-\frac{1}{3}}I_{4}\right)}^{1/2}$$

(6) $${\bar{W}}_{\mathrm{Int}}\left(I_{6},I_{3}\right)=\frac{\mu_{2}}{\gamma_{2}}\left({\left(I_{3}^{-1/3}I_{6}\right)}^{\frac{\gamma_{2}}{2}}-1\right)-\mu_{2}\ln{\left(I_{3}^{-\frac{1}{3}}I_{6}\right)}^{1/2}$$

where $\mu_{1}$, $\gamma_{1}$ and $\mu_{2}$,${\;\gamma }_{2}$ are the material parameters for the 2 families of in-plane and out-of-plane fibrils, respectively. While parameter μ controlled the stiffness of collagen fibrils, γ controlled the nonlinearity in material behaviour. ${\bar{W}}_{\mathrm{Lam}}$ and ${\bar{W}}_{\mathrm{Int}}$ will be active in Equation (4) only if $I_{4}$ and $I_{6}$ were larger than 1, indicating tension loading.

### 2.2. Data Analysis

#### Microstructure Measurements

In our earlier study, 13 strip specimens with 3.5 mm width and 20 mm length were obtained along the superior-inferior principal meridian from donor corneas aged between 21 and 90 years old. The specimens were clamped at the ends, leaving a distance of 16 mm in between, and tested under an axial tensile strain up to 8% [22]. The ethical approval to use the tissue in research was given by the University of Liverpool and donor families’ consent was obtained by the eye bank. Loading was carried out while the specimens were mounted for X-ray scanning at the Synchrotron facility in Oxford, UK to record the fibril density and angular orientation at points along the longitudinal centre line of the specimens with a uniform spacing of 0.5 mm. Each scanned point had 256 fibril density radial readings arranged with 1.4° uniform spacing, covering the whole 360°. The microstructure measurements were taken at 0%, 1.4%, 2.8%, 5.0% and 8.0% strains. Fibril distribution obtained in only the middle area of the specimens, 2mm away from the clamps, was used in the following analysis to avoid the possible stress concentration in the tissue caused by the clamps, which can affect the microstructure. Four specimens were damaged during preparation and testing, and the results of the other 9 were analysed in this study to determine the changes in fibril orientation with strain growth.

### 2.3. Normalisation

Comparisons between repeated measurements of total fibril content at each point revealed small variations (within 12.7% of the average), possibly due to measurement noise or small changes in location of scanned points from one strain level to another. As the ex-vivo tissue could not synthesise further collagen fibrils, a normalisation process was adopted whereby the total fibril content at each measurement point was equated to 1.0 in order to remove these small variations.

### 2.4. Number of Measurements of Fibril Density at Each Point

While 256 fibril density measurements provided only 128 unique values, this amount of data at each scanned point presented a challenge in the analysis of reorientation. In earlier work, we conducted an optimisation study to determine the minimum number of orientations that could be adopted without significantly affecting the outcomes of numerical models based on this microstructure data [31]. In that work, 16 orientations were selected as sufficiently representative of the microstructure data and leading to a negligible effect on numerical model results below 0.08%. This number of orientations had been adopted in this study for the same reason.

### 2.5. Fibril Reorientation

Following normalisation and reduction of the fibril density data to 16 orientations, the mean density in each orientation at each measurement point for all 9 specimens was calculated. By following this process at each strain (0%, 1.4%, 2.8%, 5%, 8%), the change in fibril density with strain growth was determined as:

(7) $$\Delta K_{\varepsilon }^{m}=K_{\varepsilon }^{m}-K_{0}^{m}$$

where $K_{\varepsilon }^{m}$ is the fibril density at orientation m (1 to 16) at strain ε∈[0%, 1.4%,2.8%,5%,8%].

### 2.6. Assumptions

The changes in angular fibril density between the 5 strain levels considered were assumed to vary linearly. This meant that the total value of $\Delta K_{\varepsilon }^{m}$ at any of the measurement points was 0 at any strain level and not only at the strain values at which microstructure data were recorded. It, therefore, ensured that the total fibril density at any point remained constant during the reorientation process.

Additionally, since specimens were aligned with the corneas’ vertical centre line, around which there was earlier evidence of symmetry in the central cornea, symmetrical fibril distribution was assumed around the specimens’ longitudinal axis. Therefore,

(8) $$\Delta K_{\varepsilon }^{m,n}=\left(\Delta K_{\varepsilon }^{m}+\Delta K_{\varepsilon }^{n}\right)/2$$

where m and n are a pair of symmetrically-located orientations around the longitudinal axis such as orientations 1 and 16, or 2 and 15. This step helped further reduce the effect of any noise in the data and reduce the number of orientations considered in the analysis from 16 to 8 unique pairs of orientations.

### 2.7. Development of Reorientation Algorithm

#### 2.7.1. Implementation of Fibril Reorientation in Numerical Models

**Figure 1:**
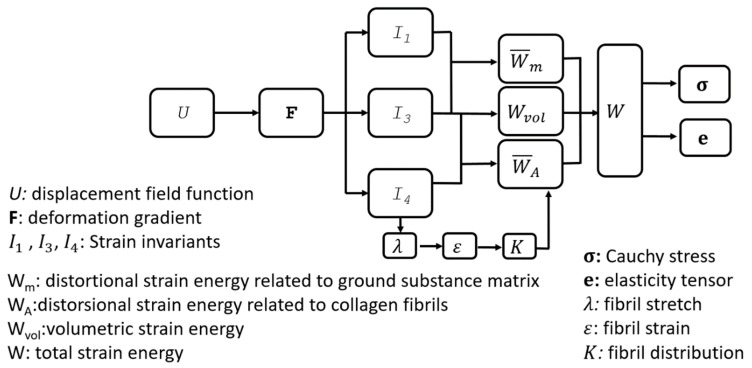
Flow chart of the method adopted to consider microstructure data in the tissue’s constitutive model and the variables involved in the material formulation. The strain value is calculated based on $I_{4}$, and fibril distribution K is altered considering the change in strain.

As explained in our earlier publication, the regional variation and anisotropy of fibril distribution across the ocular globe were introduced in the material constitutive model through fitting the microstructure data to Zernike polynomials. The polynomials were then used to estimate the fibril density and angular distribution at the integration points of the model’s finite elements [10,31], Figure 1. In this method, the fibril distribution function K stored the fibril density data as illustrated in Equation (4). The method had been modified in this study as the fibril density data, K, were allowed to vary according to the strain acting at the model’s integration points.

**Figure 2:**
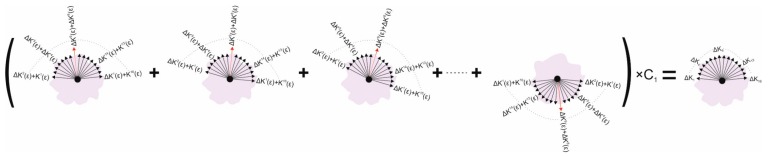
Arithmetic process to simultaneously realise fibril reorientation at multiple discretised orientations. Numbers in superscript depict the 16 discretised fibril orientations. The orientation in red is the principal strain orientation in each of the 16 calculation steps. Orientations 8 and 9 surround the loading direction in the uniaxial test. $\Delta K^{m}\left(\varepsilon \right)$ calculates the change component of fibril content caused by a strain larger than 1% at the orientation in red (ε>1%). The superscript m represents the orientation in the quantified relationship between strain and change in fibril density in the uniaxial test. The change components are added together to determine the total changes in fibril density taking place in each orientation. The fibril density related coefficient $C_{1}$ represents the averaged fibril density across the 16 orientations at the point and the correction factor that proportionally control the change in fibril density at all 16 orientations.

Considering the difference between the uniaxial loading condition, which was applied in the experiments, and the physiologic conditions where the intraocular pressure (IOP) affected all radial and tangential directions, a loading condition compensation process was needed. First, the trends of fibril reorientation were quantified by the uniaxial test analysis before being overlapped when implemented in each of the 16 fibril orientations at every integration point in the model, Figure 2. When loading in one direction in the uniaxial test, the 8th and 9th fibril orientations surrounding the loading direction showed significant increases in fibril density, while the fibril density simultaneously decreased in orientations 1 and 16. Likewise, when loading in all the 16 directions—mimicking the eye’s physiologic conditions, the strain in each orientation (for example the 8th) was treated in the same way with nearby orientations (7th and 9th) gaining higher fibril density and those perpendicular to it (1st and 16th) losing density. Considering that there should be no change in total fibril density at any point, the increases and decreases in fibril density caused by the strain change in any direction would balance each other out. Therefore, the trends of fibril reorientation were checked to ensure that the total fibril density experienced no change at all strain levels.

#### 2.7.2. Validation of Reorientation Algorithm

After deriving the fibril reorientation trends based on the X-ray scattering data, these trends were embedded in a finite element simulation of the uniaxial tissue test and the fibril reorientations predicted numerically at different strain levels were compared with those observed experimentally in order to validate the reorientation trends.

**Figure 3:**
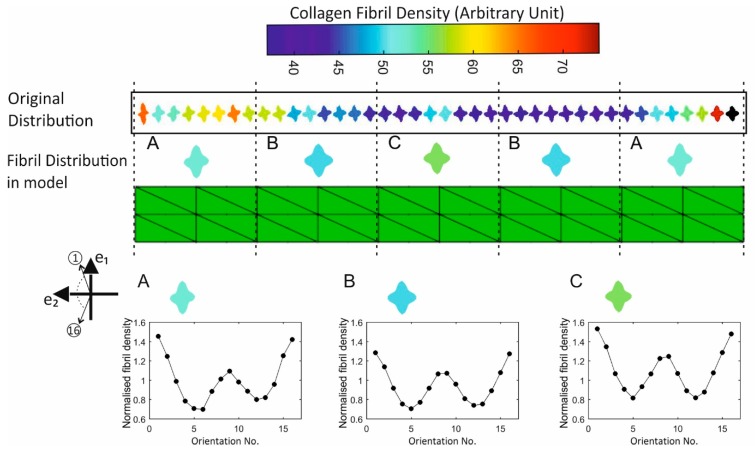
Fibril distribution applied in the strip model. The distribution was measured at 40 equally-spaced points along specimen length. These points were divided into five groups, and the average distributions were calculated for each group (separated by dash lines). The symmetrically-positioned groups were assumed to share the same fibril distribution, hence average values were calculated and applied in the model.

A numerical model was created to simulate the uniaxial test, Figure 3. The model had 80 3D continuum elements (C3D15H) arranged in 2 layers and was 14 mm in length, 3.5 mm in width and 0.5 mm in thickness. The stress-free fibril distribution as measured experimentally at 40 equally-spaced points along the specimens’ longitudinal axis was implemented in the model. This was facilitated by dividing the model into 5 regions and applying the mean of the fibril data measured at the 8 points within each region at all elements in that region. Further, since the centre of the tissue specimen coincided with the apex of the cornea, from which the specimen was extracted, mid-line symmetry was assumed. Therefore, averages of the data obtained for the 1st and 5th groups and for the 2nd and 4th groups were considered, making the model composed of only 3 distinctive regions (A, B, C in Figure 3). The standard deviation values of total fibril density were reduced by 1.8%, 6.4%, 0.5%, 0.4%, 14.9% after averaging fibril distribution in the symmetric groups. Following construction, the model was pinned at one end and a displacement causing 8% strain was applied gradually at the other end.

### 2.8. Further Numerical Assessments

#### 2.8.1. Single Element Model

**Figure 4:**
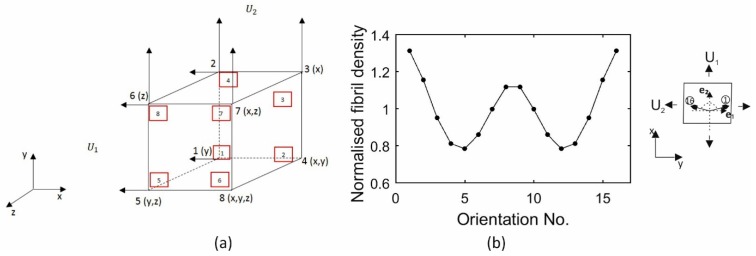
(**a**) Single element model; directions in brackets represent the boundary conditions. Node numbers are in black and numbers of integration points are in red boxes. (**b**) Initial fibril distribution assumed at all integration points.

A model made of a single element was used to provide a further assessment of the fibril reorientation algorithm and to check the ability to maintain no change in total fibril content at individual integration points as reorientation takes place with strain growth. The model was composed of an 8-noded linear, hybrid brick element (C3D8H) with dimensions 10 × 10 × 10 mm, Figure 4a. It was subjected to 3 cases of forced displacements including: (1) $U_{1}$ and $U_{2}$ displacements of 0.8 mm in X- and Y-directions at nodes 1, 2, 5, 6 and 2, 3, 6, 7, respectively, (2)${\;U}_{1}$ displacement of 0.8 mm in X-direction at nodes 1, 2, 5, 6 and $U_{2}$ displacement of 0.4 mm in Y-direction at nodes 2,3,6,7, (3) only $U_{1}$ displacement of 0.8 mm in X-direction at nodes 1, 2, 5, 6. The fibril distribution at all integration points (Figure 4b) followed that found in group B in the strip model in Figure 3.

#### 2.8.2. Full Eye Model

**Figure 5:**
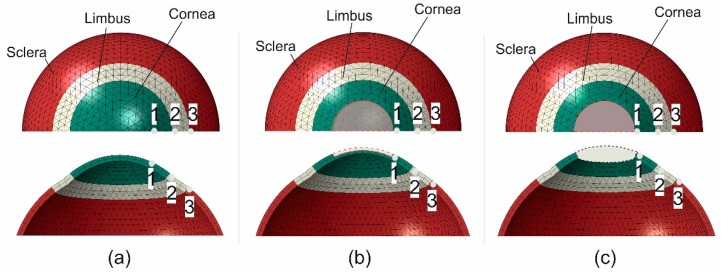
Three full eye models corresponding to (**a**) a healthy physiological state, (**b**) a simplified refractive surgery model, and (**c**) a simplified penetrating keratoplasty model. Images show front views (top) and cross-sectional views (bottom). The regions within red dash lines represent the removed tissue.

Full-eye numerical models were used to represent (1) an intact case, (2) a case with the anterior corneal stroma removed within the central 6 mm diameter zone, providing an approximate representation of tissue loss in laser-assisted in situ keratomileusis (LASIK), and (3) a case with the full corneal thickness removed within the central 6 mm diameter zone, representing penetrating keratoplasty. The first model included 12,696 15-noded continuum elements (C3D15H) arranged in 2 layers and 8 rings in the cornea and 38 rings in the sclera, Figure 5. The models used a central corneal radius, R_c_ = 7.8 mm [32,33], central corneal thickness, CCT = 0.545 mm [34], peripheral corneal thickness, PCT = 0.695 mm, limbal radius, R_L_ = 5.85 mm [35,36], scleral radius, R_s_ = 11.5 mm [37], scleral equatorial thickness, SET = 0.556 mm [38], scleral posterior pole thickness, SPT = 0.834 mm and corneal shape factor, p = 0.82. The models were loaded with a uniformly distributed internal pressure of up to 30 mmHg. This IOP value was chosen to slightly exceed the normal physiological IOP range seen in ophthalmic practice. The models’ results were used to assess the fibril reorientation in the areas close to where the tissue was removed—in particular at points 1 (para-central cornea), 2 (limbus) and 3 (anterior sclera), distances 0.41, 2.65 and 5.89 mm from the cut, respectively.

## 3. Results

### 3.1. Fibril Reorientation Trends

**Figure 6:**
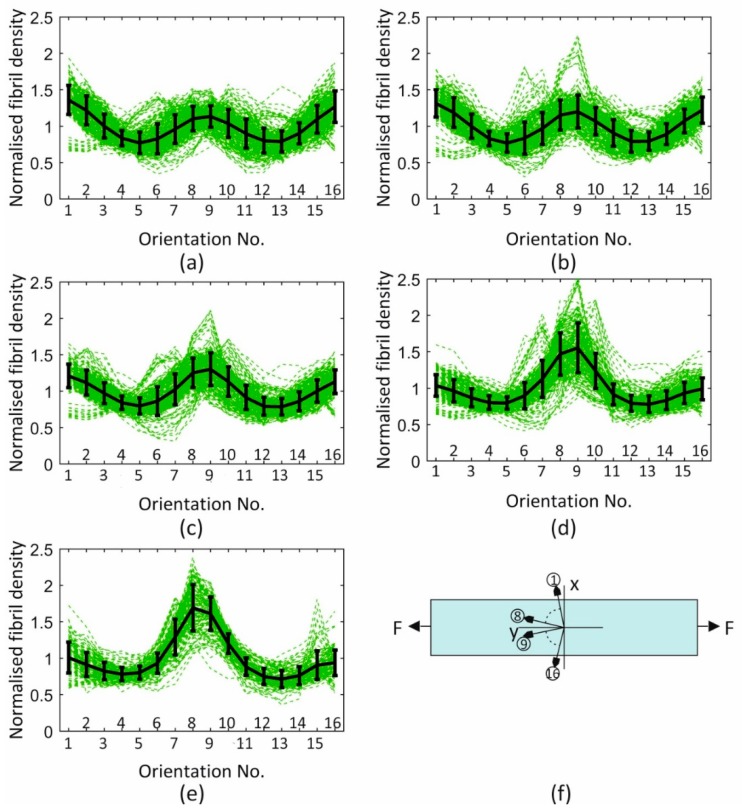
Mean and standard deviation of fibril density at all data points across specimen width recorded at s strain of (**a**) 0%, (**b**) 1.4%, (**c**) 2.8%, (**d**) 5%, and (**e**) 8%. The 16 discretised orientations considered in the figures are shown in (**f**). The orientations correspond to angle $\theta_{i}=\frac{pi}{32}+\frac{pi}{16}\left(i-1\right)$, i=1,2,⋯,16 based on the local coordinate system, and this definition was adopted in the numerical modelling. Error bars represent the standard deviation of fibril density values.

The mean values and standard deviations (SD) of fibril density recorded across the specimen width in the 16 discretised orientations and with strain growth from 0% to 8% are shown in Figure 6. As shown in the figure, orientations 8 and 9 were the closest to the axial direction, while orientations 1 and 16 surrounded the perpendicular direction. The mean values were normalised such that the average value of fibril content across all orientations was equal to 1.0 at each strain level. In addition to the expected symmetry around the specimen axial direction (between Orientations 8 and 9, and 7 and 10, etc.), there was strong evidence of increased anisotropy of fibril distribution with strain, leading to increased content in the more axial directions and hence less fibril content towards the perpendicular direction. This reorientation was slow in development until a strain of 1.4% but became increasingly evident at higher strain levels.

**Figure 7:**
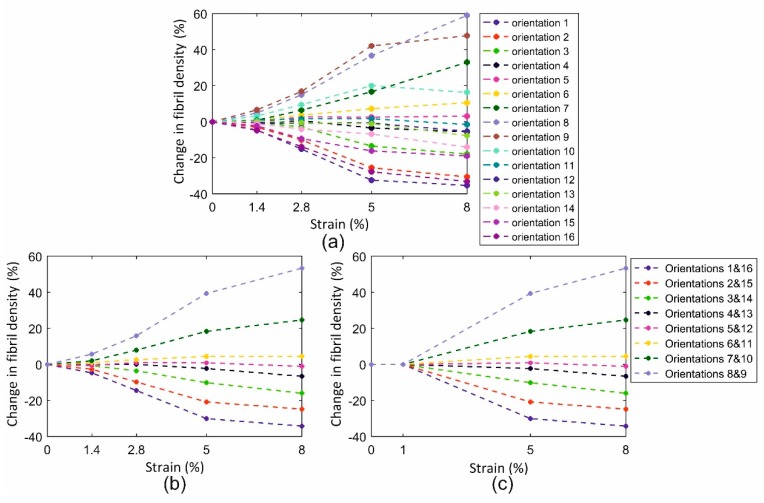
Fibril reorientation with strain increase up to 8%. (**a**) Mean results obtained experimentally for 16 orientations, (**b**) results shown while considering symmetry on opposite sides of the axial direction, (**c**) simplified reorientation behaviour to improve the stability of numerical models. (**b**) and (**c**) share the same legend.

Based on the evident symmetry of fibril density around the axial direction (mean difference between orientations on opposite sides of the axial direction was 5.3% ± 2.4%), the mean of the density data in each two opposite orientations (1 and 16, 2 and 15, …) was plotted against strain in Figure 7 and simplified to improve the stability of numerical models employing these trends of fibril reorientation. This exercise pointed at a strain of 1% as being the threshold below which reorientation was negligible (below 2%), and above which a linear reorientation took place up to 5% strain. Above this level, the change in fibril density reduced in rate by an average of 64.0% ± 26.9%.

### 3.2. Validation of Reorientation Trends against Experimental Measurements

**Figure 8:**
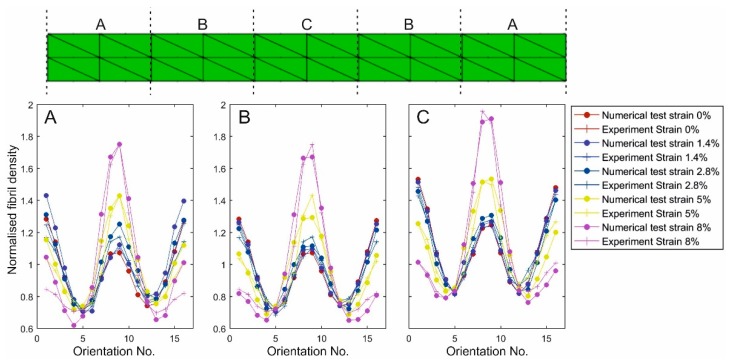
Fibril reorientation as predicted numerically and measured experimentally for the strip test. A, B and C correspond to the three zones of the strip test specimen.

The fibril reorientation trends, developed in Figure 7, were used in numerical modelling to reproduce the fibril reorientation results obtained experimentally. In the results depicted in Figure 8, the fibril density predictions showed gradual increases with strain in the axial direction. Throughout all strain levels, up to 8%, the mean errors in the three model regions A, B and C were 3.5% ± 1.6%, 3.7% ± 2.3% and 2.1% ± 1.4%, respectively.

### 3.3. Finite Element Assessment

#### 3.3.1. Single Element Model

**Figure 9:**
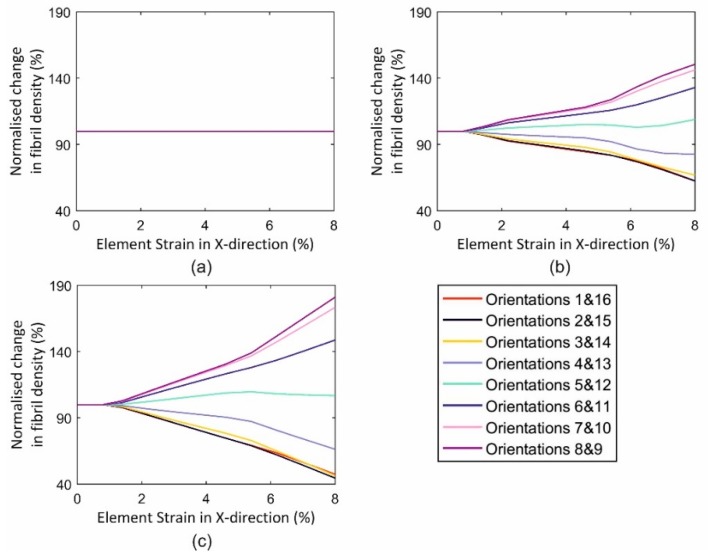
Changes in fibril density in 16 orientations in single-element models subjected to three distinctive displacement loading cases: (**a**) U1 = 0.8 mm and U2 = 0.8 mm, (**b**) U1 = 0.8 mm and U2 = 0.4 mm, and (**c**) U1 = 0.8 mm and U2 = 0.0.

The single-element model simulations showed, in a simple case, that asymmetry of loading triggered fibril reorientations that became clearer with higher strain asymmetry and higher strain levels, Figure 9. Fibril distribution underwent no change when the element was equally loaded in both the X and Y directions as there was no trigger in this case for fibril reorientation, Figure 9a. In contrast, unequal biaxial loading led to an alteration in fibril distribution, and that increased with larger strain anisotropy, Figure 9b,c. As examples, orientations 8 and 9 (closest to X direction) saw an increase in fibril density of 81.1% in case 3 (U1, U2 = 0.8 and 0.0 mm), 50.5% in case 2 (U1, U2 = 0.8 and 0.4 mm) and 0 in case 1 (U1, U2 = 0.8 and 0.8 mm), all at 5% X strain. Meanwhile, orientations 1 and 16 (almost perpendicular to X direction) underwent corresponding reductions in fibril density of 55.4%, 37.5% and 0%, respectively.

#### 3.3.2. Full Eye Model Application

**Figure 10:**
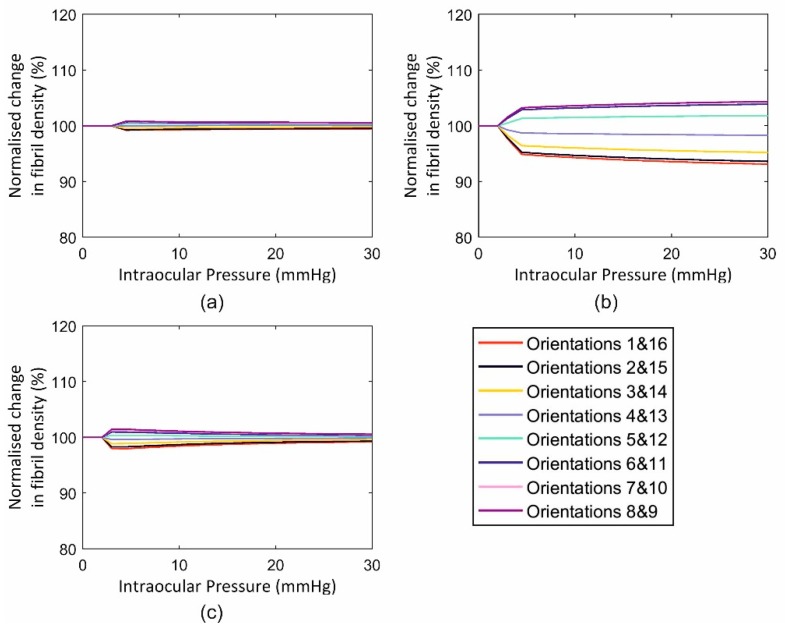
Fibril reorientation in the intact inflated ocular model under intraocular pressure up to 30 mmHg at three locations across the cornea including (**a**) element 1, (**b**) element 2 and (**c**) element 3.

**Figure 11:**
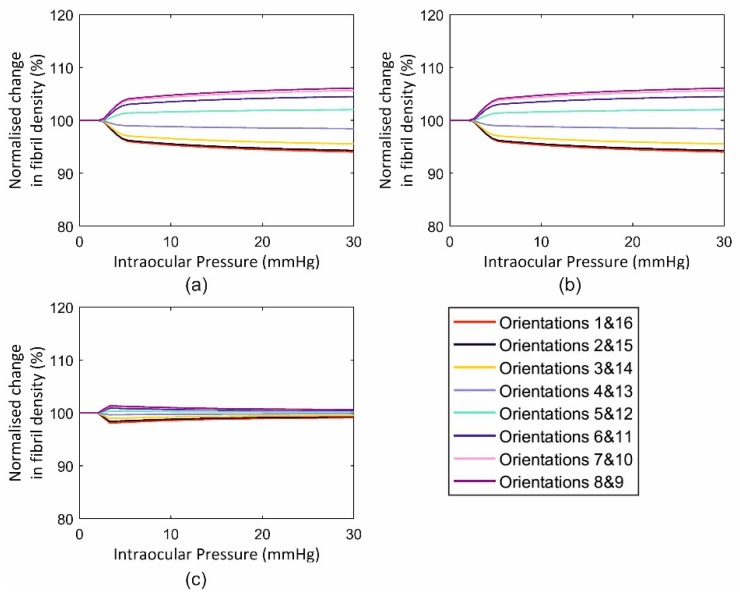
Fibril reorientation in a model of an inflated ocular globe with anterior cornea removed under intraocular pressure up to 30 mmHg at three locations across the cornea including (**a**) element 1, (**b**) element 2 and (**c**) element 3.

**Figure 12:**
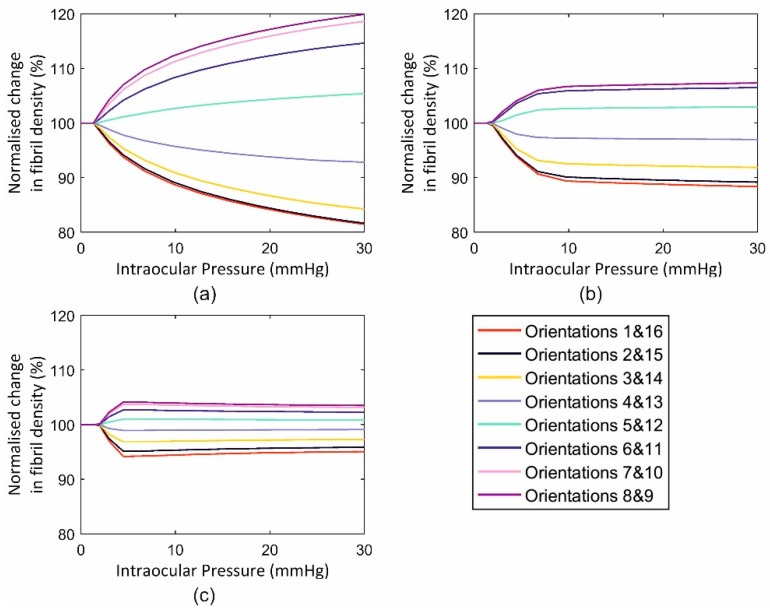
Fibril reorientation in an inflated ocular model with central corneal region removed under intraocular pressure up to 30 mmHg at three locations across the cornea including (**a**) element 1, (**b**) element 2 and (**c**) element 3.

This application included three cases with an intact globe, a globe with the anterior stroma (with half corneal thickness) removed and one with full-thickness tissue removal from the middle 6 mm-diameter zone. The behaviour of the three cases in terms of fibril reorientation under the action of an IOP of 30 mmHg is presented in Figure 10, Figure 11 and Figure 12, respectively. In all models, the circumferential strain was higher than the radial strain. However, as the ratio between the two strains was small in the intact model (max ratio = 1.0011, 1.0074, 1.0008 at points 1, 2 and 3, at the para-central cornea, limbus and anterior sclera, Figure 5), this led to slight fibril reorientation with the 8th and 9th orientations (closest to circumferential direction) increasing their fibril density by 0.6%, 0.8%, 0.6% at points 1, 2 and 3 (Figure 10) at IOP of 30 mmHg. With tissue removed at the central cornea, the strain ratio increased especially close to the edge of the removed tissue (1.0080, 1.0082, 1.0009 in second model and 1.0399, 1.0123, 1.0047 in third model), enabling larger fibril reorientations (by 6.1%, 6.8%, 1.6% in second model and 19.9%, 7.3%, 3.5% in the third model). The large difference in density changes between models was not only caused by the differences in strain ratios (circumferential/radial), but also by the growth in strain values experienced under the same IOP loading but with progressive tissue removal. As an example, the circumferential strain at point 1 (closest to the central 6mm diameter zone) increased by 3.0% and 3.2% with the partial, then full removal of the central corneal elements. In contrast, the corresponding increases in circumferential strains at the anterior sclera (farthest from the central zone) were 2.9% and 3.1%, respectively.

## 4. Discussion

Ocular diseases and refractive surgery may result in significant changes in the tissue’s microstructure, making it necessary to quantify the associated changes in mechanical behaviour under the eye’s common loads, primarily IOP [11,12,13,19,20]. In these and other studies, collagen fibrils are accepted as the main load-carrying components of the tissue with both fibril orientation and fibril density affecting its mechanical behaviour [18,26,29,39].

In this study, it is further assumed, based on experimental evidence, that while fibril content at any particular location does not change with strain growth, fibril anisotropy does. This principle of fibril reorientation has been incorporated into earlier microstructure-based constitutive models in different ways. Earlier models considered only two preferred fibril orientations whose vectors changed direction with strain growth [26,27], while later models adopted dispersion factors that changed with variations in the strain or stress regime [18,29]. Due to the lack of experimental data on fibril reorientation, earlier mathematical models were based on assumptions related to the gradual re-alignment of fibrils [18,27,28,29,40]. In this study, an alternative approach was adopted whereby the initial (unloaded) distribution of fibril density and anisotropy were obtained from X-ray scattering data of human eye globes, and the change in anisotropy was based on experimental data obtained from microstructure mapping of the tissue while being subjected to a gradual strain growth. The study benefitted from a recent publication that enabled, using tests on ex-vivo tissue, quantifying the relationship between strain and fibril reorientation [22].

The study adopted an approach whereby fibril density (content divided by tissue thickness) varied with strain in pre-determined 16 directions, instead of reorienting the directions themselves. The approach, while being simpler to implement in numerical modelling, should not lead to any significant variation in results due to the large number of orientations (16) adopted. The predictions of models, which used this approach to simulate fibril reorientation, matched closely the results of the ex-vivo tissue testing in terms of fibril distribution under different strain levels up to 8%.

Following this limited validation, the implementation of the reorientation algorithm in whole globe models simulating intact cases and cases with partial or full loss of central corneal tissue showed how the models responded to the different strain distributions in the zone immediately next to the tissue loss zone. In that region, a growth in circumferential strain caused by tissue loss led to growth in fibril density in that direction that increased with internal load, proximity to the edge of the tissue loss zone and magnitude of tissue loss. However, the growth in density of circumferential fibrils (max 6%) remained below 15% of the total collagen fibril density, which was observed in a single cornea that underwent penetrating keratoplasty (PK) and whose microstructure was characterised using the X-ray scattering technique [11]. This large difference is thought to be related to significant additional growth in overall fibril content that is thought to form part of the tissue’s response to substantial changes in the mechanical pressures it is being subjected to—similar to those experienced following PK. The inability of our material model to simulate this additional fibril growth represents a limitation of the study that can only be addressed when sufficient experimental evidence becomes available. One of the possible limitations in this study could be to the absence of in-vivo experimental evidence that shows equivalent fibril behaviour as in ex-vivo experiments because of the extreme difficulty of applying mechanical tests on a live person’s eye without risking permanent damage. Nevertheless, as the corneal tissue response to the mechanical load is mainly based on the collagen content [22], and collagen content is practically the same when comparing ex-vivo and in-vivo experimental arrangements [41], authors think that ex-vivo response-to-load (mechanical) corneal behaviour is almost equivalent to the in-vivo response. Another limitation of the study is that while it can represent fibril reorientation, it does not account for the underlying mechanism from a microscopic perspective, which is currently not well understood.

To conclude, a new method has been proposed to simulate the reorientation of collagen fibrils in corneal tissue following significant changes in strain regime. The method relied on experimental tests on ex-vivo corneal tissue subjected to uniaxial tension when being scanned using X-ray scattering. An algorithm representing the fibril reorientation trends, observed experimentally, was developed and implemented in numerical models of the cornea and whole eye globe, and shown to demonstrate reorientation of collagen towards directions of higher strain.
